# Assessing the impact of wildlife conservation areas on human well-being

**DOI:** 10.1371/journal.pone.0341609

**Published:** 2026-02-25

**Authors:** Domenic Romanello, Heriniaina M. Rakotohary, Mirana J. E. Rahariniaina, Rebecca J. Lewis

**Affiliations:** 1 Department of Anthropology, University of Texas at Austin, Austin, Texas, United States of America; 2 Mention Zoologie et Biodiversité Animale, Faculté Des Sciences, Université d’Antananarivo, Antananarivo, Madagascar; University of Jaen: Universidad de Jaen, SPAIN

## Abstract

Effective wildlife conservation is inextricably linked to the well-being of people living in and around conservation areas. Historically, conservationists have focused on a narrow range of externally defined socio-economic proxies for human well-being, failing to provide the affected population with the opportunity to assess their own life and connection to local conservation interventions. If conservation area assessments do not faithfully detect the complex and multidimensional nature of human well-being, conservation policies and practices may be misaligned with the core objectives of conservation and detrimental to the rights and livelihoods of local communities. To address this concern, we evaluated the relationship between income, multidimensional poverty, and human well-being in 594 households (1,362 individuals) bordering Kirindy Mitea National Park, Madagascar. The vast majority of local community members (86% of households) lived below the international poverty line of 2.15 US$ per day, and nearly all (95% of households) were multidimensionally ‘impoverished,’ enduring severe health, education, and living standards deprivation. Human well-being, measured using the Global Person Generated Index (GPGI), was low, with a median of 5.25 (IQR 3.25–7.00; scale range: 0.00–10.00). Higher income was associated with lower multidimensional poverty (χ² = 14.57, df = 3, p = 0.002, ε² = 0.02) and higher well-being (ρ = 0.14, p < 0.001), but multidimensional poverty was unrelated to well-being (χ² = 3.81, df = 3, p = 0.283, ε² = 0.001). Perceptions of the park were largely negative: more than 80% of households reported no benefit to income or overall well-being, and costs such as displacement and loss of access to land and resources were widely cited. Local priorities for improving well-being centered on employment, financial assistance, food security, and agricultural support, complemented by strong demand for improved healthcare, education, and community infrastructure. Our study highlights substantial human costs associated with stringent environmental protections and illustrates the importance of integrating assessments of human well-being together with socio-economic evaluations of conservation areas.

## Introduction

The relationship between socio-economic factors and human well-being continues to be a subject of significant interest among scholars, policymakers, and individuals aiming to understand the fundamental determinants of well-being [1]. Socio-economic factors, such as education level [2], access to food [3], personal health [4], and financial resources [5], influence human well-being and contribute to an overall sense of happiness [6]. However non-economic factors also play an important role (e.g., religion and spirituality [7], self-esteem [8], and social connectivity [9]). As a result, efforts to assess human well-being have evolved beyond traditional economic indicators (e.g., income, the Multidimensional Poverty Index (MPI) [10], Gross Domestic Product (GDP) [11]) to include more holistic and subjective measures that reflect how individuals define and experience well-being (e.g., the Global Person Generated Index [12,13], Gross National Happiness [14], Oxford Happiness Questionnaire [15], Quality of Life Scale [16]).

In the realm of wildlife conservation, a notable lack of widely adopted methods for assessing human well-being remains, despite calls for such evaluations [17,18]. Because protected areas (PAs) now encompass more than 16% of the world’s land surface [19], assessing their impacts on local people, particularly economically disadvantaged rural communities, is crucial [17]. However, most assessments of PAs have either overlooked human impacts entirely or relied solely on externally defined socio-economic indicators of well-being (e.g., [20–26]), but see (e.g., [27–29]). While studies often report positive results, indicating that PAs help to alleviate poverty compared to similar areas lacking conservation protection [20,23], their findings may not fully reflect the global reality. In many PAs, communities continue to endure significant hardships associated with extreme poverty [30]. Moreover, communities residing within or near these areas experience a wide range of additional effects [31,32]. Standard socio-economic metrics, such as poverty level, may fail to capture the broader ranges of both positive and negative consequences, including eviction, homelessness, landlessness, marginalization, and social disruption [33]. These complex impacts and their implications for human well-being are still inadequately captured by most conservation assessment frameworks.

Human well-being is a multidimensional construct without a single universally accepted definition [34]. Economic theory has often equated well-being with economic well-being, measured through income or consumption (e.g., [35,36]). In contrast, economist Amartya Sen conceptualizes well-being as “the gap between what a person is capable of doing and being, and what they would like to do and be; in essence, it is the gap between capability reality and expectations” ([37], page 402). Sen emphasizes that well-being derives from the freedoms individuals have to achieve valued functionings, for example being healthy, being educated, and exercising agency over one’s life [38]. Empirically, this multidimensionality has led to the development of composite indices that assess multiple domains of well-being, such as health, education, and living standards [10,39]. Within conservation, these approaches provide a means to evaluate how conservation interventions affect people’s lives, helping practitioners identify whether conservation actions support or undermine human well-being in communities that depend on wildlife [40].

Happiness, commonly defined as an enduring emotional state characterized by positive affect [41,42], differs from human well-being due to its narrower scope. Empirical research typically operationalizes happiness through measures of life satisfaction or joy (e.g., [43–46]). These measures capture a single aspect of subjective experience, whereas well-being frameworks incorporate multiple objective and subjective domains such as material conditions, health, and social relationships [47]. Within the context of conservation, happiness can be understood as one component among many components that contribute to overall well-being. By examining both happiness and well-being, conservationists can gain deeper insights into the diverse ways conservation efforts affect human lives [17,48].

The Global Person Generated Index (GPGI) has been applied in conservation contexts as a capabilities-based measure of well-being that centers on individuals’ self-identified priorities [12,27,49]. Measures, such as happiness [15], life satisfaction [16], and quality of life [46], are conceptually and methodologically distinct from the GPGI, which uniquely requires respondents to identify and evaluate dimensions of well-being [12,13] that are personally meaningful. The GPGI aligns with theoretical approaches that conceptualize well-being in terms of capabilities, providing an empirical means of assessing how individuals define and evaluate their own well-being [27]. The GPGI captures both socio-economic and non-socio-economic dimensions of well-being by allowing respondents to define and evaluate the domains of life they consider most important [27]. This measure complements traditional poverty and happiness indicators by revealing how conservation interventions influence well-being in locally meaningful terms [27,50]. However, empirical data on the effects of PAs on human well-being remains limited (see [27–29,51]), reflecting the relatively recent adoption of participatory frameworks like the GPGI that enable community members to evaluate their own lives and their relationship to conservation efforts [17]. Incorporating such stakeholder-based measures of well-being into conservation assessments is critical because conservation policies that appear successful when judged by poverty reduction alone may nevertheless fail to improve overall well-being.

Madagascar presents a compelling context for evaluating how new approaches to conservation assessment perform in complex socio-ecological systems. In response to environmental crises, the country rapidly expanded its PA network [52], but the effect of this expansion on human well-being is not yet understood. Between 2003 and 2016, the extent of protected land quadrupled, positioning Madagascar as a model for other countries striving for wildlife conservation [52,53]. However, the impact of this expansion on human well-being remains largely unknown. Despite the growth of PAs, threats to biodiversity have persisted [54]. Habitat loss, hunting, and other pressures have continued to cause environmental degradation even within designated conservation zones [55–58]. One major contributing factor is widespread poverty – 80% of Malagasy citizens live in extreme income poverty, surviving on less than 2.15 US$ per day [59]. Economic hardship exacerbates environmental pressures as coping strategies often involve environmentally harmful practices [60]. For instance, child malnutrition, food insecurity, and poor health, are all recognized drivers of hunting and the consumption of protected and endangered species in Madagascar [61–68]. As such, a nuanced understanding of the relationship between PAs and the well-being of surrounding communities is essential to enhancing the effectiveness and sustainability of conservation efforts in the country.

To understand the relationship between PAs and human well-being, we examined both the socio-economic impacts and broader human dimensions associated with a specific PA in Madagascar. We first hypothesized a negative relationship between income and poverty [69], predicting that as household incomes decreased, levels of deprivation increased across multiple dimensions of poverty including health, education, and living standards. Next, we hypothesized a negative relationship between poverty and human well-being [49], and predicted that level of well-being is associated with increased incomes and reduced deprivation across these same dimensions. We then hypothesized that participants perceive their socio-economic status and overall well-being to be influenced by the presence of the PA [27], predicting that these perceived impacts are positive, specifically with respect to income, health, education, living standards, and overall well-being. Finally, we compared perceived impacts of the PA with observed outcomes and conducted a community-based needs assessment to identify local priorities for well-being.

## Materials and methods

### Study site

**Fig 1:**
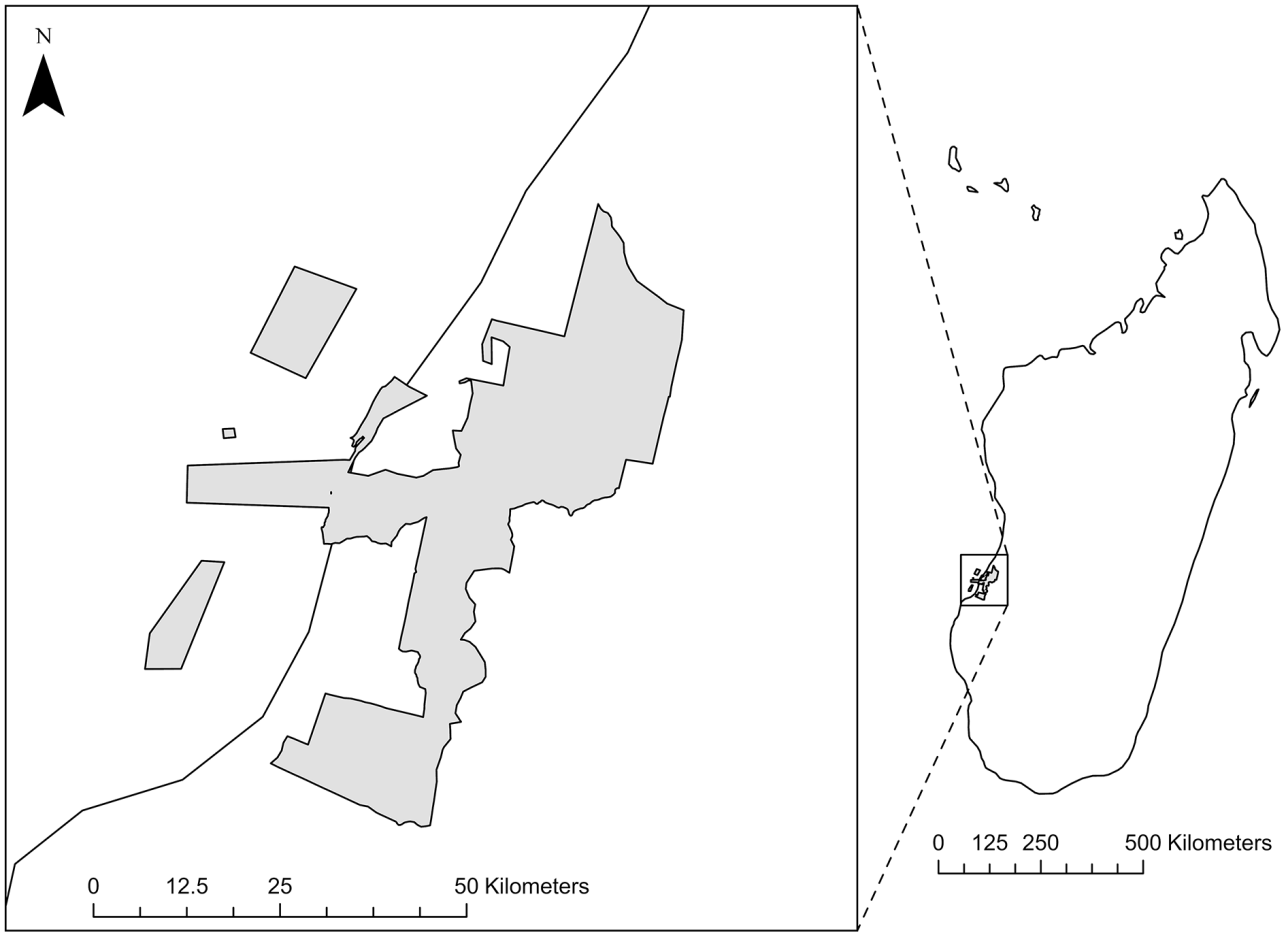
Study site map of Kirindy Mitea National Park, Madagascar. The main panel shows the boundary of Kirindy Mitea National Park (shaded grey), located along the west coast of Madagascar. The inset map illustrates the location of the park within Madagascar, indicated by a dashed locator box. Park boundary data were obtained from Madagascar Protected Areas [77]. The Madagascar outline was sourced from Natural Earth (public domain).

We surveyed households on the border of Kirindy Mitea National Park (KMNP; IUCN category II; Fig 1) in the southern Menabe region of Madagascar [70]. Established on 18 December 1997, the park was created to protect endemic fauna (known species richness: birds – 132, reptiles – 31, bats – 13, lemurs – 8, amphibians – 6, tenrecs – 5, carnivores – 3), and flora (mangrove forest, arid spiny thicket, dry deciduous forest, grassland) [71]. KMNP is large (area: 156,183 ha, elevation: 0–250 m), spans marine and terrestrial environments, and is contained within the broader Belo-sur-Mer – Kirindy-Mitea UNESCO Biosphere Reserve (est. 2016, 625,050 ha), an area designated to promote wildlife conservation and sustainable development [72]. The local climate is characteristic of the sub-arid southwest of Madagascar, with 95% of rainfall occurring between November and April [71]. The Menabe region is home to over 700,000 people [73], with approximately 150,000 residing in the administrative district that contains KMNP [74]. Although the predominant cultural identity in the Menabe region is Sakalava [75], a variety of other cultural groups are also present, partly due to recent migration from the southwest [76]. Many residents around KMNP self-identify as Sakalava-Vezo (primarily fishers) and/or Sakalava-Masikoro (primarily farmers/herders) [61,75]. Livelihood strategies include agriculture, fishing, illegal hunting and logging, pastoralism, selling goods, shipping and transportation (by animal cart, boat, or vehicle), and wage labor such as seasonal salt harvesting and limited work in conservation and ecotourism (D. Romanello, personal observation). Severe poverty, food insecurity, and malnutrition are widespread [57,61].

### Survey administration

We conducted data collection over four consecutive months (April–July 2023) in seven rural communities bordering KMNP (1,362 individuals, 594 households), using the local Sakalava Malagasy dialect. We collaborated with community leaders to develop a complete list of households in each study community and approached all households for participation. This approach ensured that our survey was effectively a census of all households in the study communities rather than a random sub-sample, thereby maximizing representativeness and minimizing selection bias. Each survey began with a greeting, self-identification of male or female head of household, and documentation of household members (defined as individuals who regularly reside and share meals at that location). If all household members were present, interested in participating, and willing to provide the necessary verbal consent, assent, and parental permission, the survey proceeded. We collected the following data for each household member: sex, relationship to the head of household, years of schooling, and school attendance status. We also collected body weight and height data, which are necessary for quantifying malnutrition and calculating MPI deprivation scores. Because malnutrition thresholds are age dependent, we also collected data on age and date of birth. Then, household members were informed that the remainder of the survey would be completed solely with the head of household through structured and semi-structured interviews. These interviews were conducted inside or near participant’s homes, based on their preference, to ensure privacy. Questions were presented using an iPad and responses were recorded using the Kobo Toolbox app.

### Ethical note

The University of Texas at Austin Internal Review Board reviewed and approved all research methods, including the use of verbal consent, assent, and parental permission, as well as the procedures for documenting and witnessing consent (IRB STUDY #00001646). In accordance with the IRB-approved protocol, all consent, assent, and parental permissions were provided verbally and audio recorded to document and witness participation. Research assistants trained in ethical protocols served as witnesses and confirmed each participant’s verbal consent during the recording. The recruitment period for this study began 1 April 2023 and ended 31 July 2023. Before conducting this work, we acquired the requisite permits and approval from the Ministry of Environment and Forests and Madagascar National Parks at the national, regional, and local levels with the facilitation of the Madagascar Institute for the Conservation of Tropical Environments (Permit #040/23/MEDD/SG/DGGE/DAPRNE/SCBE.Re). We also met with local leaders in each of the study communities to inform them of the details of the study, answer questions, and ask whether their community would be interested in participating. Leaders of all communities approved and permitted us to commence with our research. People who did not wish to participate in our study, and those unable to consent were excluded. Exclusion criteria for this study included anyone who was imprisoned, or who self-identified as suffering from (a) a severe mental or physical disability/illness (including Alzheimer’s, dementia, etc.), (b) a severe developmental disability, (c) a severe psychiatric illness, suffering from any alcohol or drug dependencies, or (d) a recent traumatic event (e.g., a death in the family, domestic abuse). All potential study participants were read a consent form in Sakalava Malagasy. Study participants aged 18 years or older provided verbal consent. For children older than 6 years of age and younger than 18 years of age, a parent provided informed verbal consent in addition to the child’s assent. For children aged 0–6 years, a parent provided informed verbal consent, but the child’s assent was not required. Self-identified heads of household younger than 18 years of age in the study population provided verbal assent, and parental permission was not required. We did not document written consent for any of the participants because (1) literacy rates are low, and (2) signatures would have been inherently incriminating given the sensitive subject matter of the surveys. Consent, assent, and parental permission were provided verbally and audio recorded as approved by the IRB. We informed all participants of the purpose of our research and the potential benefits, risks, and discomforts associated with participation in our study. All participants were informed that their participation was voluntary, and that they could withdraw at any point during the study, end a survey early, or skip any questions they did not feel comfortable answering. To ensure anonymity, names and other personally identifiable information were not collected.

### Measuring income, poverty, and human well-being

To measure daily per capita household income, we asked each head of household to estimate the average daily income derived from all household members’ combined earnings, including salaries, sales, and wage labor. This total was then divided by the number of household members. We assessed poverty using the Multidimensional Poverty Index (MPI) [10], a composite measure based on ten indicators (S1 Table). All ten indicators of the MPI fall into the three broad dimensions of poverty: 1) health (nutrition and child mortality), 2) education (years of schooling and school attendance), and 3) living standards (cooking fuel, sanitation, drinking water, electricity, housing, and assets). The MPI generates a deprivation score from 0–1. For each indicator, households receive a score of 1 if they are deprived (e.g., no access to electricity) and 0 if not. These scores are then multiplied by the assigned weight of each indicator (e.g., 1 * 1 ÷ 18; note: 1 ÷ 18 is the assigned weight of the electricity indicator). The weighted values are summed to generate the household’s final MPI deprivation score. Interpretation of MPI score is as follows: ‘vulnerable to poverty’ (≥0.20), ‘impoverished’ (≥0.33), and ‘severely impoverished’ (≥0.50).

We measured human well-being using the Global Person Generated Index [12,13,26], a participant-centered tool that differs from the MPI by allowing individuals to define well-being based on their own priorities. The GPGI has been successfully applied in several cultural contexts, including prior studies in Madagascar [12,27,49]. Participants were asked to identify five dimensions they considered most important to their well-being (e.g., agriculture, community relations, religion). For each selected dimension, participants first assigned a performance score on a five-point scale, ranging from (0 (very bad, as you fear)’ to 4 (very good, as you want). Then, they distributed 10 pebbles among the five dimensions to indicate the relative importance of each. The GPGI score for each dimension was calculated by dividing the performance score by four, then multiplying by the number of pebbles assigned to that dimension. For example, if a participant rated a dimension with a performance score of 3 (‘good, but not as good as you want’), and ‘spends’ 5 pebbles, the GPGI score for that dimension is calculated as follows: (3 ÷ 4) * 5. The final GPGI score is the sum of the scores across all five dimensions and ranges from 0 to 10, with higher scores indicating greater overall well-being. To assess local perceptions, we analyzed both household views of the park’s impacts on income, poverty, and well-being, and community-defined priorities for improving well-being, drawing on open-ended survey responses and domains generated during the GPGI exercise. Responses were thematically coded into domains and analyzed by frequency, relative importance, and performance.

### Data analysis

We used R Statistical Software v.4.5.1. [78] to analyze the relationship between income, poverty, and human well-being in communities near KMNP, and to assess the park’s impact on these dimensions. Because these data did not meet the assumptions of parametric models, all group comparisons and correlations were conducted using nonparametric tests (Kruskal–Wallis, Wilcoxon rank-sum, Spearman’s rank, Fisher’s exact). We first summarized household demographic and socio-economic characteristics (sex, age, household size, income, MPI deprivation scores, and GPGI scores) using descriptive statistics reported as median (IQR). To assess the local relevance of MPI indicators, we asked heads of household whether they agreed or disagreed that each indicator was essential to household well-being. Responses were summarized as proportions of agreement/disagreement for each indicator.

We then tested our first hypothesis by comparing household income across MPI deprivation categories (‘not impoverished’, ‘vulnerable to poverty’, ‘impoverished’, and ‘severely impoverished’) using a Kruskal–Wallis test, followed by post hoc Wilcoxon rank-sum tests. Household income was log-transformed to emphasize proportional differences at low-income levels, a standard approach in poverty and well-being research (e.g., [79]). We next examined associations between logged income and household deprivation across each MPI indicator using Wilcoxon rank-sum tests, including asset-specific analyses.

To test our second hypothesis, we evaluated the association between logged household income and GPGI scores using Spearman’s rank correlation. We compared GPGI scores across MPI deprivation categories using a Kruskal–Wallis test, and across individual MPI indicators using Wilcoxon rank-sum tests. Additional Wilcoxon rank-sum tests were used to evaluate associations between asset ownership and GPGI scores. To test our third hypothesis, we compared participants’ perceptions of the impact of KMNP on local incomes, MPI indicators, and overall well-being to observed outcomes. We used Wilcoxon rank-sum tests to compare logged income, MPI deprivation scores (including the living standards dimension), and GPGI scores between households that perceived positive impacts and those that did not. For categorical MPI indicators related to education and health (e.g., years of schooling, school attendance, child mortality, malnutrition), we applied Fisher’s exact tests to evaluate associations with reported park impacts.

Finally, we assessed community priorities for improving well-being using two approaches. First, heads of household answered the open-ended question: “What should be done to improve life for you and your family?” Responses were thematically coded into domains and ranked by frequency of mention. Second, we analyzed the domains generated through the GPGI, thematically coded these in the same way, and plotted mean performance against mean relative importance.

## Results

### Household characteristics

The study participants consisted of 1,362 individuals from 594 households, with 56% (768) identifying as female and 44% (594) identifying as male. The median age of participants was 20 years (IQR 8–33), with age ranging from zero to 100 years. Median household size was 2 members (IQR 1–3). Daily per capita household income ranged from 0.00 to 11.50 US$ based on a conversion rate of 1 MGA is 0.00023 US$. Median income was 1.15 US$ (IQR 0.58–1.38 US$) and a large majority of households (86%) lived below the international poverty line of 2.15 US$ per day, indicating widespread ‘extreme income poverty.’

MPI deprivation scores ranged from 0.17 to 1.0 (scale range: 0.00 to 1.00), with a median of 0.67 (IQR 0.61–0.78). The majority of households (86%) were classified as ‘severely impoverished’ (score ≥ 0.50), while nearly all households (95%) met the threshold for being ‘impoverished’ (score ≥ 0.33). A small portion were considered ‘vulnerable to poverty’ (score ≥ 0.20) and less than 1% were not deprived (score < 0.20).

### MPI health and education dimension

The health dimension of the MPI includes two indicators: child mortality and nutrition. More than one-quarter (29%) of households were deprived of nutrition, indicating at least one household member was malnourished based on body mass index (Table 1). In terms of child mortality, 12% of households had experienced the death of a child within the five years preceding the survey. The education dimension also consists of two indicators: years of schooling and school attendance. A large majority (85%) were deprived of years of schooling, with no member over the age of 10 having completed at least six years of education. Additionally, nearly half (47%) of households with school-aged children were deprived of school attendance, meaning at least one child aged 6–14 was not enrolled in school.

**Table 1:** Community-identified priority domains for improving household well-being near Kirindy Mitea National Park, Madagascar.

Rank	Priority domain	Number of mentions
**1**	Livelihood activities or jobs (includes agriculture)	323
**2**	Money and wealth	261
**3**	Food	172
**4**	Agriculture	129
**5**	Asset ownership	97
**6**	Home ownership and quality of the home	45
**7**	Education	18
**8**	Clean drinking water	17
**9**	Health	15

### MPI living standards dimension

The living standards dimension of the MPI comprises six indicators: cooking fuel, sanitation, drinking water, electricity, housing, and assets. All households were deprived in the cooking fuel, sanitation, and drinking water indicators, as they relied on wood or charcoal for cooking, lacked access to a private flush toilet or latrine, and did not have access to safe drinking water. Nearly all households (96%) were deprived in the electricity indicator due the absence of electricity in their homes. In terms of housing, 85% of households were classified as deprived because their homes were constructed from rudimentary materials. For the asset indicator, 91% of households were deprived because they did not own a car or truck and lacked more than one of the following items: radio, television, telephone, computer, animal cart, bicycle, motorbike, or refrigerator.

### Human well-being

GPGI scores ranged from 0.00 to 10.00 (scale range: 0.00–10.00), with a median of 5.25 (IQR 3.25–7.00). The most frequently reported domains of human well-being reported by heads of household (53% male, 47% female), included assets, family, livelihood activities or jobs, agriculture, food, home ownership and quality of the home, money and wealth, health, and education (S2 Table). Three additional domains were identified by less than 20% of respondents, while 16 domains were cited by less than 5%.

### Local relevance of the MPI

Over 98% of heads of household agreed or strongly agreed that avoiding undernutrition and child mortality, along with ensuring school attendance, access to electricity, home building materials, and safe drinking water, were essential to the well-being of household members. Additionally, 95% of heads of household agreed or strongly agreed that access to a flush toilet or latrine was essential. By contrast, 12% of heads of household disagreed or strongly disagreed that avoiding cooking with wood or charcoal was essential to the well-being of household members. In terms of asset ownership, over 98% of heads of household agreed or strongly agreed that ownership of a radio, refrigerator, telephone, and television was essential to the well-being of household members. Regarding other assets, 92% of heads of household agreed or strongly agreed that ownership of a bicycle was essential, 93% for a car, 93% for a motorbike, 95% for a computer, and 97% for an animal cart.

### Income and poverty

Logged household income significantly differed across MPI categories (‘not impoverished’, ‘vulnerable to poverty’, ‘impoverished’, and ‘severely impoverished’; χ² = 14.57, df = 3, p = 0.002, ε² = 0.02). Households classified as impoverished or severely impoverished had significantly lower logged incomes than households classified as not impoverished or vulnerable to poverty (W = 4857, p < 0.001, r = −0.14). When the MPI was disaggregated into its 10 indicators, households deprived of assets (W = 8549, p < 0.001, adjusted p < 0.001, r = −0.19), electricity (W = 3071, p < 0.001, adjusted p = 0.002, r = −0.14), and years of schooling (W = 16,698, p = 0.003, adjusted p = 0.006, r = 0.12), had significantly lower logged incomes than non-deprived households. No significant difference in logged incomes were found between deprived and non-deprived households for the remaining indicators. Animal cart (W = 22,760, p < 0.001, adjusted p < 0.001, r = 0.15), radio (W = 6,453, p = 0.015, adjusted p = 0.034, r = 0.10), telephone (W = 36,071, p < 0.001, adjusted p < 0.001, r = 0.20), and television (W = 5,445, p = 0.017, adjusted p = 0.034, r = 0.10) ownership were associated with higher logged incomes. No significant differences in income were found between households with and without other assets.

### Poverty and human well-being

Logged household income was positively associated with GPGI scores (ρ = 0.14, p < 0.001). GPGI scores did not differ across MPI categories (‘not impoverished’, ‘vulnerable to poverty’, ‘impoverished’, and ‘severely impoverished’; χ² = 3.81, df = 3, p = 0.283, ε² = 0.001). When the MPI was disaggregated into its 10 indicators, households that had experienced the death of a child within the five years preceding the survey had significantly lower GPGI scores than non-deprived households (W = 13,719, p = 0.001, adjusted p = 0.007, r = −0.13). No significant difference in GPGI scores were found between deprived and non-deprived households for the remaining indicators. Radio (W = 7,514, p = 0.005, adjusted p = 0.02, *r* = 0.11) and telephone (W = 35,593, p < 0.001, adjusted p = 0.002, *r* = 0.15) ownership was associated with higher GPGI scores. No significant difference in GPGI scores were found between deprived and non-deprived households for the other assets.

### Impact of kirindy mitea national park on poverty and human well-being

**Fig 2:**
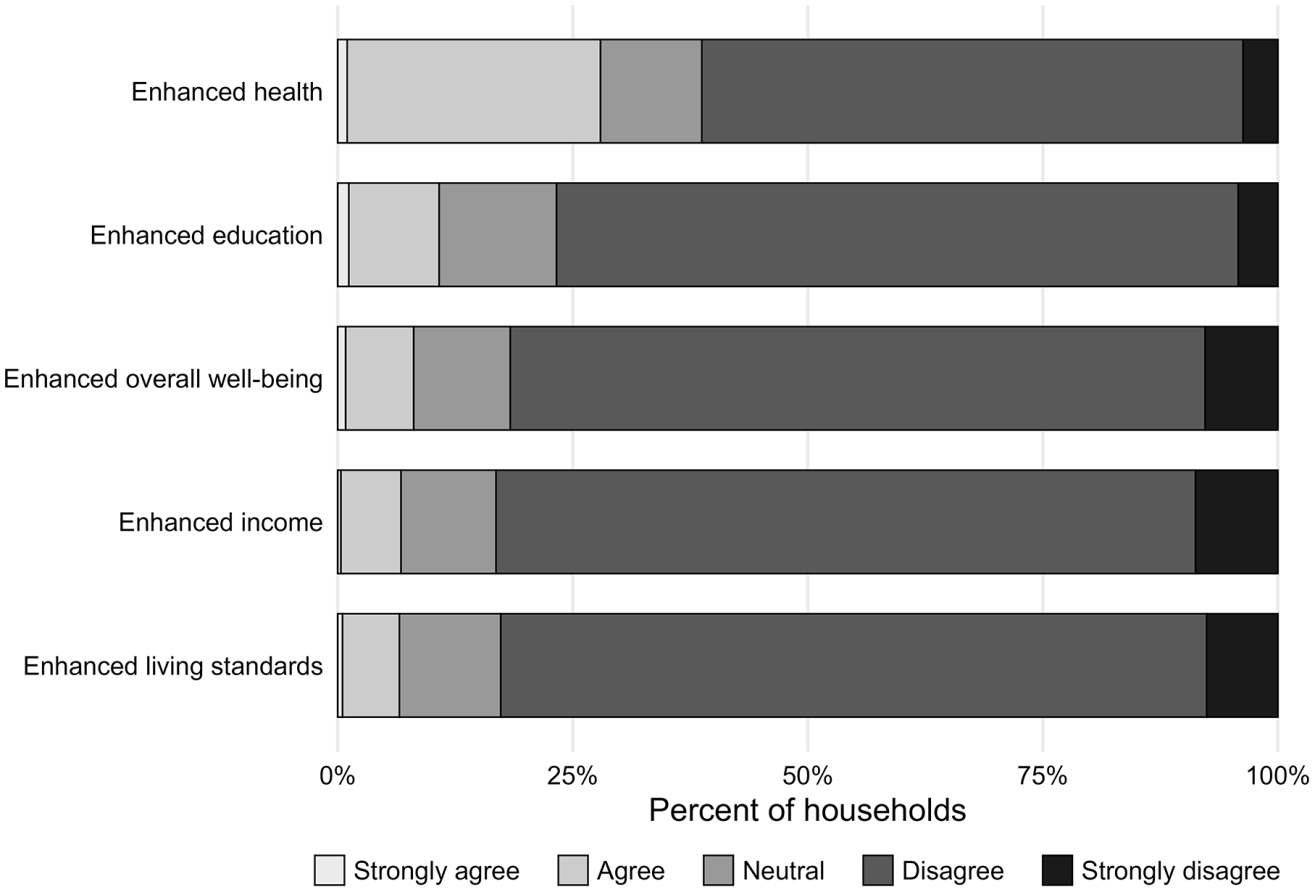
Perceived impacts of Kirindy Mitea National Park on household well-being. Stacked bar plots show the percentage of households that strongly agreed, agreed, disagreed, strongly disagreed, or were neutral regarding whether the park enhanced health, education, living standards, income, or overall well-being.

Most households disagreed or strongly disagreed that the park enhanced their income (83%), education (77%), living standards (83%), or overall well-being (82%) (Fig 2). Limited employment opportunities within and around the park meant that socio-economic benefits were unevenly distributed. Some individuals secured full-time positions, others obtained temporary or part-time work, but the vast majority did not gain employment. As one participant explained, “our life became good when the park was established because some jobs were available, and my son works there.” Some people had a problem “because they did not know where to find work or money.” Those who did find employment reported being able to afford food, improve their homes, pay rent, cover children’s school fees, and access healthcare. However, such benefits were limited by the low availability of paid work, both within the park and in the surrounding area.

Development projects associated with KMNP, such as school construction, seed distribution, or well installation, were seldom mentioned and often criticized for their inadequacy. For example, one participant shared, “they gave us peanut seeds, but it was during the bad year with insufficient rainfall, and when we planted them, they were destroyed because the land was too dry.” Wells installed as part of development initiatives were contaminated with various dangerous substances like salt, making the water unsafe and its taste unpleasant. Even in communities where schools were built by park management, many people struggled to afford the associated cost.

Perceptions were somewhat favorable for health, with more than one-quarter (28%) of households agreeing or strongly agreeing, but the majority (61%) still disagreed (Fig 2). In this context, participants most commonly cited health benefits such as access to fresh air, rainwater, and traditional medicinal resources. As one participant explained, “if the forest was destroyed, the land would become dry, and rainwater would go away because it depends on the forest. The agriculture needs rainwater, and after it has rained, we can cultivate our land and make money.” Among the various domains of human well-being identified by participants, KMNP was found to contribute to only one domain: forest and environment (S2 Table). For this domain, the mean performance score was described as ‘good but not as good as you want.’ Only 1% of households identified this domain, and those who did generally agreed that KMNP contributed positively to it. However, participants generally disagree that KMNP impacts performance in all other well-being dimensions.

Respondents who agreed the park positively impacted their income had significantly higher logged income than those who disagreed (W = 12,661, p = 0.0019, r = 0.14). Households who agreed the park positively impacted education were significantly less likely to be deprived in years of schooling compared to those who disagreed (p < 0.001, OR = 3.65, 95% CI [1.93, 6.78]), whereas no significant association was found for school attendance among households with school-aged children (p = 0.21, OR = 2.88, 95% CI [0.53, 14.65]). Agreement that the park improved health was not associated with child mortality deprivation (p = 0.24, OR = 1.49, 95% CI [0.79, 2.97]), nor malnutrition deprivation (p = 0.25, OR = 0.79, 95% CI [0.52, 1.20]). Households who agreed the park positively impacted living standards had lower deprivation scores in the MPI living-standards dimension than those who disagreed (W = 7177.5, p < 0.001, r = 0.33). Households who agreed the park positively impacted overall well-being had significantly higher GPGI scores compared to those who disagreed (W = 15,743, p < 0.001, r = 0.18).

The primary cost of KMNP reported by local communities was displacement, manifested through forced evictions, the loss of land and resources, and restricted access to areas within the newly established park boundaries. Prior to its designation, the forest served as a critical resource base, providing food, grazing land, hunting grounds, and farmland. With the creation of the park, inhabitants were evicted and required to seek new livelihoods beyond the so-called “forbidden forest.” Study participants offered firsthand accounts of these displacements. One said, “my ancestor lived in the forest during the wet season and returned to the village in the dry season. He grew corn and peanuts there. After the park was established, that became impossible.” Another remarked, “life before the park was good. We could access the forest freely, but now it is protected, it belongs to the government.” Beyond material losses, displacements also had socio-emotional repercussions. One participant shared that his father “became sad after he left the forest,” expressing uncertainty about whether “the establishment of the park has destroyed his life or not.” The effects of displacement were not limited to those who were forcibly relocated. The broader community experienced hardships due to access to farmland and forest resources being limited. One participant explained, “After the park was established, it was no longer possible to farm in the forest. Agriculture became forbidden, and life got worse.” Some even advocated for re-access to the forest for subsistence agriculture, viewing the park more as a constraint than a benefit.

### Needs assessment

**Fig 3:**
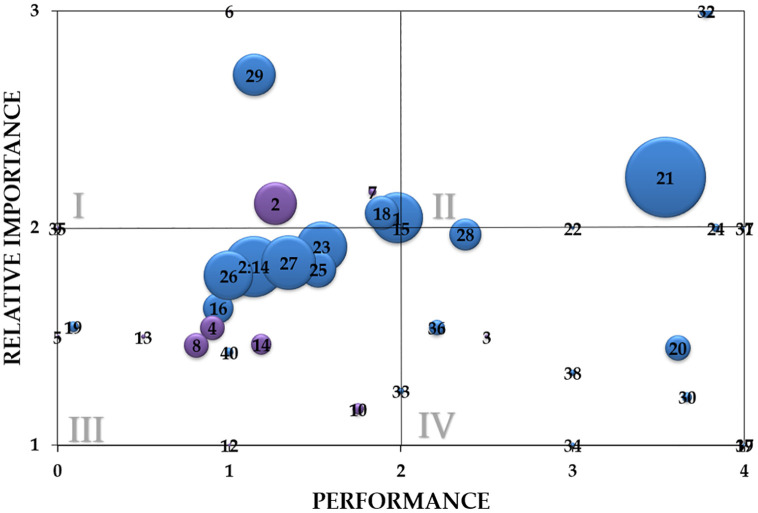
Domains of human well-being generated by heads of household living near Kirindy Mitea National Park, Madagascar. *x*-axis: mean performance score of each domain (0 – very bad as you fear, 1 – bad but not as bad as you fear, 2 – medium, 3 – good but not as good as you want, 4 – very good as you want), *y*-axis: mean relative importance of domains (measured by asking heads of household to distribute 10 pebbles across their top five domains). Point size is determined by the percentage of heads of household that self-generated each domain, with larger points signifying more popular domains, and smaller points signifying less popular domains. The four quadrants in the plot signify the following: I (domains that, on average, are deemed relatively more important than domains in quadrants III and IV, with low to medium performance), II (domains that, on average, are deemed relatively more important than domains in quadrants III and IV, with medium to high performance), III (domains that, on average, are deemed relatively less important than domains in quadrants I and II, with low to medium performance), IV (domains that, on average, are deemed relatively less important than domains in quadrants I and II, with medium to high performance). Blue points – 1: agriculture, 2-14: household assets, 15: belonging, 16: clean drinking water, 17: discussions with neighbors, 18: education, 19: electricity, 20: extended family, 21: family (children, grandchildren, parents, spouse), 22: fishing, 23: food, 24: forest and environment, 25: health, 26: home ownership and quality of the home, 27: livelihood activities or jobs, 28: livestock, 29: money and wealth, 30: neighbors, 31: parental relationship with children, 32: religion, 33: romantic partner, 34: salt harvesting, 35: security, 36: selling goods, 37: singing, 38: tobacco, 39: truth, 40: water. The household assets domain (2-14) was segregated into its component sub-domains. These sub-domains are represented with purple points – 2: animal cart, 3: appliances, 4: clothing, 5: computer, 6: cookware and tableware, 7: firearms, 8: furniture, 9: jewels, 10: phone, 11: radio, 12: soap, 13: television, 14: transportation.

Our needs assessment identified nine priority areas for future investment aimed at enhancing human well-being around KMNP. These domains reflect areas where communities face significant challenges and due to their high perceived importance, represent strong opportunities for targeted interventions. Livelihood activities or jobs emerged as a top priority (Fig 3, Table 1). Participants emphasized the urgent need for more and better job opportunities. Employment within the conservation sector was seen as particularly promising, offering both economic and environmental benefits. Agriculture was another commonly cited concern, with farmers highlighting losses due to drought and heavy rainfall. Climate related pressures have reduced yields and driven cropland expansion and deforestation regionally. Farmers called for improved irrigation, essential equipment like animal carts and plows, and expanded access to farmland, including “permission to cultivate forested areas.” Money and wealth were also frequently mentioned with many study participants expressing that increased financial resources would enable them to invest in agricultural practices or start a small business. Some requested direct monetary assistance to meet basic needs, particularly food. Many spoke of hunger and malnutrition, requesting staples like rice, beans, corn, and cooking oil to help diversify diets and address acute food insecurity. Access to assets was another key domain. Animal carts were especially valued for their role in farming and transporting goods. Other desired assets included beds, bicycles, cars, clothes, motorbikes, soap, and fishing nets. Home improvements were a major concern, with many expressing a desire for sturdier housing materials such as stone walls and sheet metal roofs, critical in a region prone to cyclones. Our fieldwork confirmed widespread housing damage, including collapsed or missing walls, roofs, and floors. Finally, participants stressed the importance of community-level infrastructure, including schools, sanitary wells, and hospitals. Access to healthcare, clean water, and education remains limited due to both distance and cost. Many also pointed out the need for financial support to afford healthcare services and school fees.

Livestock was not identified as a priority domain. While frequently mentioned, our study participants generally rated their performance higher in this area than others. Nevertheless, conversations revealed strong underlying demand for livestock, particularly small livestock such as goats, pigs, poultry, and sheep. In contrast, cattle were viewed as risky due to the threat posed by *dahalo* – cattle thieves who are known for violent crimes including kidnapping and murder. Community members expressed a desire for greater access to small livestock as a means to improve nutrition and establish more reliable income sources. Importantly, participants emphasized that improving local well-being would reduce pressure on KMNP. As one individual explained, “if the people have work, no one will destroy the forest.” While there was broad support for wildlife conservation, many felt it came at a personal cost. Ultimately, participants advocated for investments that would allow them to improve their own lives and the lives of their families, while also protecting the forest ecosystem upon which they depend.

## Discussion

### Conceptualizing human well-being

Material well-being, which is derived from access to economic resources and provision of basic needs, is distinct from the broader concept of well-being, which captures the material, psychological, and social experiences of life [34,39,47]. Although income was associated with higher well-being, the lack of association between multidimensional poverty and the GPGI highlights that material deprivation alone does not fully account for how individuals evaluate their lives. Participants prioritized domains such as family relationships, spirituality, and community, indicating that non-material dimensions are central to their well-being, consistent with evidence from well-being research in low and middle income countries [12,27,49,80]. Human well-being, therefore, cannot be inferred from material conditions alone and these findings support theoretical arguments that subjective and relational dimensions are fundamental components of human well-being [81].

### Severe poverty and low human well-being in conservation areas

Across many rural conservation areas, households experience severe deprivations in their material and subjective well-being [30–32]. People living around KMNP have extremely low incomes, consistent with a previous study of a nearby community [57]. The proportion of households experiencing severe income poverty near KMNP exceeds the Madagascar national average (80% [59]). Although the GPGI is not a widely adopted global standard for measuring well-being, its participant-driven approach offers unique, context-specific insights. Due to limited use, comparative data from other regions is scarce. Nonetheless, GPGI scores from KMNP were similar to those reported in other PAs in Madagascar [27]. The mean GPGI score in our study (5.14) was notably lower than those reported in Bangladesh (7.17), Ethiopia (6.15), and Thailand (5.24) [12], though these comparisons are based on an older study. More recent research indicates that well-being in Madagascar remains lower than in these countries, suggesting the disparity likely continues [82]. However, cross-country comparisons should be interpreted with caution, especially when different well-being metrics are used. For our study population, the GPGI is particularly valid, as it captures individual priorities and provides a direct reflection of local perceptions of well-being [12]. The prevalence and severity of poverty and low human well-being underscore a persistent paradox: PAs designed to preserve healthy ecosystems are often inhabited or bordered by communities facing chronic deprivation.

### Perceptions of conservation benefits and costs

Conservation areas provide a range of benefits to local communities, primarily through the delivery of ecosystem services [83,84]. These include cultural services (e.g., aesthetic, and spiritual values), provisioning services (e.g., food, fuel wood), regulatory services (e.g., climate regulation, water purification), and supporting services (e.g., nutrient cycling, soil formation) [85]. Ecosystem services are often the most commonly recognized benefits associated with PAs [86], and in the case of KMNP, they were also identified as the main benefit by the local study population. Non-ecosystem services benefits from KMNP were rarely reported by study participants. Few participants agreed or strongly agreed that the park had improved their household’s income, education, living standards, or overall well-being. Notably, perceptions of positive impacts on income, education, living standards, and overall well-being were more common among households in comparatively stronger socio-economic position, aligning with research showing that conservation benefits are unevenly distributed, accruing disproportionately to wealthier households [31,32]. When conservation benefits are narrowly concentrated, the negative effects of strict conservation policies can be exacerbated [30,87]. Consequently, the perceived benefits of KMNP are largely limited to ecosystem services shared by the broader community, while direct socio-economic advantages are experienced by only a small portion of the population, highlighting the complexities of linking conservation efforts to development outcomes in rural Madagascar [88].

The primary cost of KMNP raised by participants was displacement, including forced evictions, loss of land, and restricted access to natural resources. Exclusionary conservation practices have caused severe socio-economic and emotional hardship globally [89,90]. In Madagascar, as elsewhere, the creation of strict PAs often curtailed long-standing practices such as shifting cultivation, cattle grazing, and forest product collection, eroding both livelihoods and cultural ties to the land [91,92]. Without providing viable livelihood alternatives, strict protection policies risk exacerbating poverty, reducing human well-being, and fueling resentment toward conservation [31–33,89,93,94]. Together, these findings reinforce evidence that conservation strategies are most effective when they address, rather than restrict, local livelihoods, even as they deliver ecosystem services shared by the broader community.

### Community needs and pathways forward

Addressing poverty in conservation areas requires meeting the priorities communities themselves identify before expecting conservation goals to succeed [31,95,96]. Local priorities centered on livelihood opportunities such as stable employment, financial assistance, agricultural support and land access, and improved food security. Participants also highlighted the importance of non-material domains of well-being such as family cohesion, social relationships, and religious and spiritual life. These findings align with broader research showing that rural communities conceptualize well-being as multidimensional, encompassing material security and relational or cultural dimensions [27], and underscore calls for conservation to respond to community-defined priorities rather than externally imposed development metrics [97]. Efforts to achieve higher-order goals, such as wildlife conservation, are unlikely to succeed until basic material needs are met, a principle long recognized in human motivation and development theory [98,99]. For KMNP, securing land access and tenure rights is fundamental to sustaining local agriculture and reducing household food insecurity [100–102]. Complementary strategies could include the introduction of direct economic support through mechanisms such as conservation basic income schemes [103–105], conditional cash transfers [106], or livestock provisions [57], which provide pathways to diversify livelihoods and decrease dependence on increasingly restricted forest resources. Equally important are investments in infrastructure and basic services including clean water, durable housing, medical care, and education, which address structural barriers that undermine both well-being and conservation legitimacy [96,107]. Conservation outcomes in KMNP must be pursued in tandem with poverty alleviation and improvements to local well-being.

### The relationship between poverty and human well-being in conservation areas

While poverty and human well-being are related, one cannot substitute for the other, because each is necessary to reveal complementary insights into human outcomes in conservation contexts [17]. Higher incomes were associated with reduced poverty and greater human well-being around KMNP; however, no relationship was found between multidimensional poverty and human well-being. This finding may be explained by (a) the relative homogeneity of multidimensional deprivation across households in poor communities [108–110]; (b) the conceptual distinction between material or objective well-being and holistic or subjective well-being [38]; (c) the fact that while income can reduce poverty and improve well- being, material deprivation alone does not necessarily correspond to how people perceive and evaluate their lives [111]; and (d) the unequal emotional weight of specific deprivations, such as child mortality, which can have disproportionate influence on overall well-being, masking relationships with other poverty indicators [112,113]. These dynamics highlight why the relationship between poverty and well-being is context-dependent and why conservation efforts that focus solely on income or poverty reduction risk overlooking the factors that ultimately determine how people experience conservation outcomes.

## Conclusion

Conservationists worldwide have long emphasized that wildlife protection measures should contribute to human well-being while posing minimal risk to rural impoverished communities [114,115]. However, traditional conservation assessments rely heavily on strictly socio-economic measures of human well-being [116], assuming that improved economic status equals a better life (e.g., [26]). We identify two key issues with this approach. First, socio-economic measures are typically externally defined, rather than stakeholder-defined, and therefore not necessarily relevant to human well-being across different cultures and contexts [12]. Second, these measures omit non-socio-economic determinants of human well-being and may not accommodate individuals who are, for example, materially prosperous but otherwise unhappy or unwell [1]. Our findings illustrate the importance of distinguishing poverty (or material/objective well-being) from the broader concept of human well-being (or holistic/subjective well-being) in conservation contexts [117]. Our findings also highlight that conservation success cannot be achieved through biodiversity protection alone but must also address the material and non-material priorities that local communities themselves identify as central to a good life. Securing land access, strengthening livelihoods, and investing in infrastructure and basic services are not peripheral to conservation but essential conditions for its effectiveness [118]. The case of KMNP illustrates that when well-being is ignored, conservation risks eroding the very social foundations it depends upon. By integrating community-defined measures of well-being into conservation planning, KMNP and other strict conservation areas can move beyond exclusionary practices toward approaches that are just and socio-ecologically sustainable.

## Supporting information

S1 Data Anonymized household survey data collected from communities near Kirindy Mitea National Park, Madagascar. (XLSX)

S1 Table Deprivation criteria for each indicator of the Multidimensional Poverty Index (MPI). (DOCX)

S2 Table Domains of human well-being identified by study participants outside Kirindy Mitea National Park, Madagascar. (DOCX)
