# Supplementary material for: Assessing the impact of wildlife conservation areas on human well-being
Source: PLoS One. 2026 Feb 25;21(2):e0341609. doi: 10.1371/journal.pone.0341609 (PMC12935242; doi:10.1371/journal.pone.0341609)
Supplement: S1 Table — (DOCX) [file pone.0341609.s002.docx]

**Assessing the impact of wildlife conservation areas on human well-being.**

**Short Title:** Conservation and human well-being.

**Authors:** Domenic Romanello^1*^, Heriniaina M. Rakotohary^2^, Mirana J. E. Rahariniaina^2^, Rebecca J Lewis^1^

**Author Affiliations**

1. Department of Anthropology, University of Texas at Austin, Austin, Texas, USA.
2. Mention Zoologie et Biodiversité Animale, Faculté Des Sciences, Université d'Antananarivo, Antananarivo, Madagascar.

**Corresponding Author:** Domenic Romanello: [romanello@utexas.edu](mailto:romanello@utexas.edu)

**Table S1** Deprivation criteria for each indicator of the Multidimensional Poverty Index (MPI).

| **Indicator** | **Deprivation definition** |
| --- | --- |
| **Nutrition** | Any household member under 70 years of age is malnourished based on body-mass index. |
| **Child mortality** | Any child belonging to the household died in the five-year period preceding the survey. |
| **Years of schooling** | No household member aged 10 years or older has completed six or more years of schooling. |
| **School attendance** | Any household members between 6 and 14 years of age are not attending school. |
| **Cooking fuel** | The household cooks with dung, wood, or charcoal. |
| **Sanitation** | The household lacks access to its own flush toilet or latrine. |
| **Drinking water** | The household lacks access to safe drinking water within a 30-minute round-trip walk from home. |
| **Electricity** | The household lacks electricity. |
| **Housing** | The home is made of rudimentary materials. |
| **Assets** | No household member owns a car or truck, or one or more of the following items: radio, television, telephone, computer, animal cart, bicycle, motorbike, refrigerator. |
