# Supplementary material for: Assessing the impact of wildlife conservation areas on human well-being
Source: PLoS One. 2026 Feb 25;21(2):e0341609. doi: 10.1371/journal.pone.0341609 (PMC12935242; doi:10.1371/journal.pone.0341609)
Supplement: S2 Table — (DOCX) [file pone.0341609.s003.docx]

**Assessing the impact of wildlife conservation areas on human well-being.**

**Short Title:** Conservation and human well-being.

**Authors:** Domenic Romanello^1*^, Heriniaina M. Rakotohary^2^, Mirana J. E. Rahariniaina^2^, Rebecca J Lewis^1^

**Author Affiliations**

1. Department of Anthropology, University of Texas at Austin, Austin, Texas, USA.
2. Mention Zoologie et Biodiversité Animale, Faculté Des Sciences, Université d'Antananarivo, Antananarivo, Madagascar.

**Corresponding Author:** Domenic Romanello: [romanello@utexas.edu](mailto:romanello@utexas.edu)

**Table S2** Domains of human well-being identified by study participants outside Kirindy Mitea National Park, Madagascar.

| **Domain** |  | **Percentage of Households that Identified the Domain** | **Mean Performance** | **Mean Impact** | **Mean Relative Importance** |
| --- | --- | --- | --- | --- | --- |
| **Agriculture** |  | 46% | 1.98 | 2.19 | 2.05 |
| **Asset Ownership** |  | 63% | 1.14 | 2.29 | 1.82 |
|  | **animal cart** | 31% | 1.27 | 2.22 | 2.11 |
|  | **appliances** | <1% | 2.50 | 2.00 | 1.50 |
|  | **clothing** | 10% | 0.90 | 2.45 | 1.54 |
|  | **computer** | <1% | 0.00 | 1.50 | 1.50 |
|  | **cookware and tableware** | <1% | 1.00 | 1.00 | 3.00 |
|  | **firearm** | 1% | 1.83 | 2.00 | 2.17 |
|  | **furniture** | 10% | 0.81 | 2.52 | 1.46 |
|  | **jewels** | <1% | 0.00 | 2.00 | 2.00 |
|  | **phone** | 2% | 1.75 | 2.50 | 1.17 |
|  | **radio** | 1% | 1.40 | 2.00 | 3.60 |
|  | **soap** | <1% | 1.00 | 3.00 | 1.00 |
|  | **television** | <1% | 0.50 | 2.00 | 1.50 |
|  | **transportation** | 6% | 1.19 | 2.14 | 1.47 |
| **Belonging** |  | <1% | 2.00 | 3.00 | 2.00 |
| **Clean drinking water** |  | 16% | 0.94 | 2.25 | 1.63 |
| **Discussions with the neighbors** |  | <1% | 4.00 | 3.00 | 1.00 |
| **Education** |  | 20% | 1.89 | 2.21 | 2.07 |
| **Electricity** |  | 2% | 0.09 | 2.36 | 1.55 |
| **Extended Family** |  | 11% | 3.61 | 2.27 | 1.45 |
| **Family: Children, Grandchildren, Parents, Spouse** |  | 56% | 3.54 | 2.32 | 2.23 |
| **Fishing** |  | <1% | 3.00 | 1.00 | 2.00 |
| **Food** |  | 45% | 1.54 | 2.28 | 1.91 |
| **Forest and Environment** |  | 1% | 3.83 | 4.33 | 2.00 |
| **Health** |  | 23% | 1.52 | 2.23 | 1.81 |
| **Home ownership and quality of the home** |  | 42% | 1.00 | 2.30 | 1.78 |
| **Livelihood activities or jobs** |  | 52% | 1.35 | 2.22 | 1.84 |
| **Livestock** |  | 16% | 2.38 | 2.24 | 1.97 |
| **Money and wealth** |  | 32% | 1.15 | 2.26 | 2.71 |
| **Neighbors** |  | <1% | 4.00 | 3.00 | 1.00 |
| **Parental relationship with children** |  | <1% | 4.00 | 3.00 | 2.00 |
| **Religion** |  | 3% | 3.78 | 2.61 | 3.00 |
| **Romantic partner** |  | 1% | 2.00 | 2.25 | 1.25 |
| **Salt harvesting** |  | 0% | 3.00 | 3.00 | 1.00 |
| **Security** |  | <1% | 0.00 | 2.00 | 2.00 |
| **Selling goods** |  | 4% | 2.21 | 2.25 | 1.54 |
| **Singing** |  | <1% | 4.00 | 1.00 | 2.00 |
| **Tobacco** |  | 1% | 3.00 | 2.33 | 1.33 |
| **Truth** |  | <1% | 4.00 | 2.00 | 1.00 |
| **Water** |  | 1% | 1.00 | 2.86 | 1.43 |

**Note:** Mean performance score of each domain (measured as: 0 – very bad as you fear, 1 – bad but not as bad as you fear, 2 - medium, 3 – good but not as good as you want, 4 – very good as you want), mean impact score of each domain (measured by asking the following: "I would now like to ask your level of agreement with the following statement: the establishment and management of Kirindy Mitea National Park has contributed to your performance score in this domain. Do you 1- strongly disagree? Do you 2- disagree? Are you 3- neutral? Do you 4- agree? Or do you 5- strongly agree?"), mean relative importance of each domain (measured by asking heads of household to distribute 10 pebbles across their top five domains).
